# Brain injury unmasking Ehlers-Danlos syndromes after trauma: the fiber print

**DOI:** 10.1186/s13023-016-0428-9

**Published:** 2016-04-22

**Authors:** Claude Hamonet, Daniel Frédy, Jérémie H. Lefèvre, Sacha Bourgeois-Gironde, Jean-David Zeitoun

**Affiliations:** Department of Physical and Rehabilitation Medicine, Hôtel Dieu Hospital, APHP, Paris, France; Department of Medicine, University Paris-East-Créteil (UPEC), Créteil, France; Department of Neuroradiology, Saint-Anne Hospital, Paris, France; Department of General and Digestive Surgery, Saint-Antoine Hospital, APHP, Paris, France; University Paris VI, Paris, France; Laboratory of Modern Economics, Paris II University, Paris, France; Department of Gastroenterology and Nutrition, Saint-Antoine Hospital, APHP, 284, rue du Faubourg Saint-Antoine, 75012 Paris, France; Department of Proctology, Croix Saint-Simon Hospital, Paris, France

**Keywords:** Ehlers-Danlos syndrome, Brain trauma, Brain injury

## Abstract

**Background:**

The role of physical trauma in the onset of symptoms in Ehlers-Danlos syndrome (EDS) has never been characterized. We sought to search and describe brain lesions EDS patients also having personal history of physical trauma. We systematically performed brain magnetic resonance imaging in a first cohort of patients with a hypermobility type of EDS which described the onset of their disease or its worsening after a physical trauma. Unexpected yet consistent findings that were thought to be related to the reported traumas led to perform brain imaging in all subsequent patients with similar symptoms regardless of a history of trauma and to search for a prior trauma by active questioning.

**Results:**

Fifty-nine patients were recruited and analyzed, among which 53 (89.8 %) were women. Overall, 26 (44.1 %) reported a personal history of physical trauma. Six signs pertaining to subcortical lesions and affecting white matter tracts were identified. Those included lesions of the reticular formation, the two lenticular nuclei, the corpus callosum and the arcuate fasciculus. Thirty-six patients (61.0 %) had at least 5 of the 6 imaging signs. In case of a trauma before 18, patients had significantly more lesions of the reticular formation (100 % vs. 50 %; *p* = 0.0035).

**Conclusions:**

Patients with EDS, hypermobility type, were found to have consistent and specific brain lesions involving white matter tracts. Moreover, the record of a physical trauma in a substantial proportion of cases suggests that these lesions could be post-trauma consequences. Therefore, physical trauma could be a triggering factor in EDS.

## Background

Ehlers-Danlos syndromes (EDSs) are a heterogeneous group of connective tissue disorders mainly characterized by joint hypermobility, skin hyperextensibility and tissue fragility [1]. Despite the fact that the first clinical description dates back more than a century [2, 3], EDSs are still poorly known, their genetic background is largely ignored and their pathophysiology is unclear. Additionally, we dramatically lack efficacious treatments. Last, although the Villefranche’s classification is still the benchmark [4], their nosology and semiology are still a matter of debate [5]. For instance, we recently described that the frequency and type of digestive symptoms in EDS were much more common and different than previously reported [6]. Yet, many subjective symptoms of EDS are not understood, in particular those that could be related to the nervous system.

Anecdotal observation collated in the frame of our routine clinical activity suggested that several patients reported their symptoms being unmasked or worsened following a physical traumatism. This primarily appeared counter-intuitive for a genetic disease to be triggered by a possible injury. Yet, we aimed to search and evaluate possible lesions of the central nervous system (CNS) and magnetic resonance imaging (MRI) examinations were performed that showed abnormalities which were thought to be attributable to numerous symptoms described by patients. Subsequently, MRIs were also performed in other patients with similar subjective signs that could be linked to CNS lesions but with no recorded history of trauma and those showed brain lesions analogous to the ones that had been previously identified, suggesting that minor traumas could have occurred but gone unnoticed during childhood. We herein report the results of this series of 59 patients.

## Methods

All patients described in the current report were recruited from February 2010 through August 2013 following a regular visit in our tertiary center dedicated for EDS. They were clinically assessed at least once by a single expert practitioner in EDS (CH) and were formally diagnosed has having a hypermobility type of EDS according to Villefranche criteria [4]. All of them had joint hypermobility, skin hyperextensibility and/or smooth, velvety skin, and joint disorders. Joint hypermobility was specifically assessed using the Beighton scale [7], and all recruited patients had a score above the threshold of 5 points. All patients had a familial history of EDS, either for their ascendants or descendants.

This study was conducted in accordance with the French bioethics laws which do not require any approval by an ethic committee for clinical and imaging examinations as long as they are performed in the frame of standard cares. All patients provided written informed consent.

### Sample 1: patients with symptoms and personal history of physical trauma

Initially, a first subset of 25 patients was identified for their claim of exacerbation of neurologic symptoms following a physical trauma (hit, car accident, physical abuse, etc.), especially because some of them needed objective data proving their disorders as they were suing for compensation. The reported signs included proprioceptive disorders, dystonia, altered vigilance and sleep disorders, pain syndrome, dysautonomic syndrome, chilliness, sweating, impaired thermoregulation. Therefore, they were referred for brain imaging in order to search for a cause to their complaints.

### Sample 2: subsequent patients with no spontaneously reported history of physical trauma

Following the intriguing findings of multiple, seemingly specific and systematic abnormalities in patients of the sample 1, subsequent patients (*n* = 34) seen in consultation with similar clinical symptoms but no reported history of physical trauma were also systematically referred for brain imaging in the attempt of searching for analog brain injury.

### Magnetic resonance imaging

MRI scans were all performed by the same investigator (DF) according to the same techniques. MRI evaluations were made with a 1.5 tesla machine (General Electric®) following Flair, T2*, Fiesta, and 3D-SPGR T1 sequences. Tensor diffusion imaging was also part of the MRI assessment.

### Statistical analysis

Categorical variables are described as frequencies and percentages, and continuous variables are described as the means ± standard deviation. Comparisons among groups were performed using the Chi-squared or Fisher’s exact test for discrete variables and by unpaired t-tests or the Wilcoxon rank-sum test for continuous variables. All tests were two sided with a level of significance set at *p* < 0.05. All statistical tests were performed using SAS software version 9.3; SAS Institute Inc., Cary, NC, USA.

## Results

Overall, fifty-nine patients were included in the present series. General characteristics are shown in Table 1.

**Table 1:** General characteristics, MRI findings of the overall sample of patients

Characteristics	*n* (%)
Patients’ Characteristics	
Female gender	53 (89.8 %)
Age	38.53 ± 15.5
Right handed	28 (45.5 %)
Previous history of trauma	26 (44.1 %)
Mean age of first trauma	25.92 ± 16.04
MRI findings	
Lesions of the reticular formation	48 (81.4 %)
Lesion in Fiesta sequence	22 (37.3 %)
Lesion in DTI sequence	42 (71.2 %)
Both reticular lesions	16 (11.9 %)
Lesion in the lenticular nuclei	55 (93.2 %)
Lesion in the anterior part	54 (91.5 %)
Lesion in the posterior part	54 (91.5 %)
Both lesions	53 (89.8 %)
Lesions of the corpus callosum	58 (98.3 %)
Injury of the arcuate fasciculus	49 (83.1 %)
Bilateral sub-cortical atrophy	49 (83.1 %)
Leucopathia	10 (16.9 %)
Numbers of signs on MRI	
6	4 (6.8 %)
5	32 (54.2 %)
4	13 (22.0 %)
3	8 (13.6 %)
2	0 (0 %)
1	0 (0 %)
0	2 (3.4 %)

### Magnetic resonance imaging findings

Six main criteria pertaining to subcortical lesions were identified. Those lesions affected white matter tracts, essentially made of myelin coated axons.

**Fig. 1:**
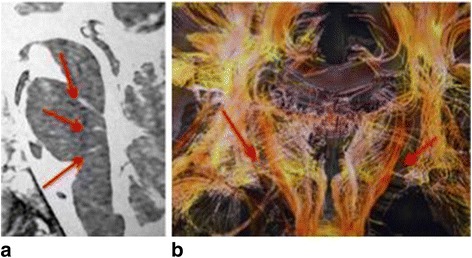
**a** Post-traumatic aspect of the reticular formation in a Fiesta sequence equally weighing T1/T2 ratios. This Figure comes from a 34 years-old women reporting the onset of vigilance and sleep disorders following a car accident ten years before. **b** Rarefaction of vertical cortical-spinal fibers of the reticular formation in 3D sequence
- The second criterion was a similar lesion pattern in the two lenticular nuclei. Tridimensional sequences showed a quasi-symmetrical bilateral rarefaction of fibers crossing the anterior [Fig. 2a] and posterior [Fig. 2b] parts of the two lenticular nuclei. This fiber tract appeared cut off after a cerebra trauma. Since these fibers spread over the two frontal lobes and the basal ganglia, their rupture and rarefaction tended to be associated with attentional and motivational troubles.

- The first criterion was the presence of lesions of the reticular formation, which is a posterior medial cord extending downwards the whole length of the brainstem. It is a network of longitudinal nerve fibers that regulates several autonomic functions such as consciousness and sleep. Two types of lesions that were thought to be specific were identified with respect to this first criterion. The first lesions were observed in the medial part of the reticular formation and perpendicular to the back of the floor of the 4^th^ ventricle. As shown in a Fiesta sequence equally weighing T1/T2 ratios, the lesions exhibited a post-traumatic aspect (Fig. 1a). The second lesion affecting the reticular formation consisted in a bilateral rarefaction of vertical cortical-spinal (spinal-thalamic) fibers. Those fibers plunge within the lenticular nuclei and go through the two cerebral peduncles. Besides their sensitive function, they collaterally support the reticular formation. Tridimensional diffusion tensor imaging (DTI) revealed the fibers rarefaction (Fig. 1b).

**Fig. 2:**
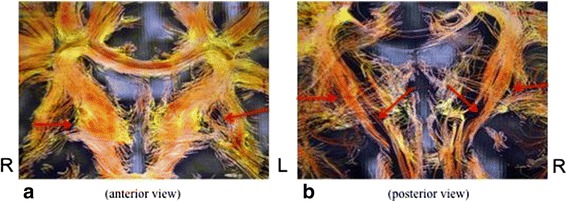
**a** and **b** Bilateral rarefaction of fibers respectively crossing the anterior and posterior parts of the two lenticular nuclei. Those Figures pertain to a 42 years-old women reporting the onset of dystonia after she had a car accident when she was 38
- The third retained criterion was the presence of lesions of the corpus callosum exhibiting a post-traumatic aspect. Bidimensional-DTI showed a gloss drop of the corpus callosum which was bilateral and more pronounced in its posterior part [Fig. 3a]. This aspect could be correlated with functional impairment, consistently with previous reports. Indeed, lesions or dysmorphia of corpus callosum have been shown to be associated with extreme plasticity in auditory and motor scaffolding [8] and tactile anomia [9] (Fig. 3a). 3D-DTI reveals in addition a lateral [Fig. 3b] or bilateral [Fig. 3c] rarefaction of white fibers in this structure.

**Fig. 3:**
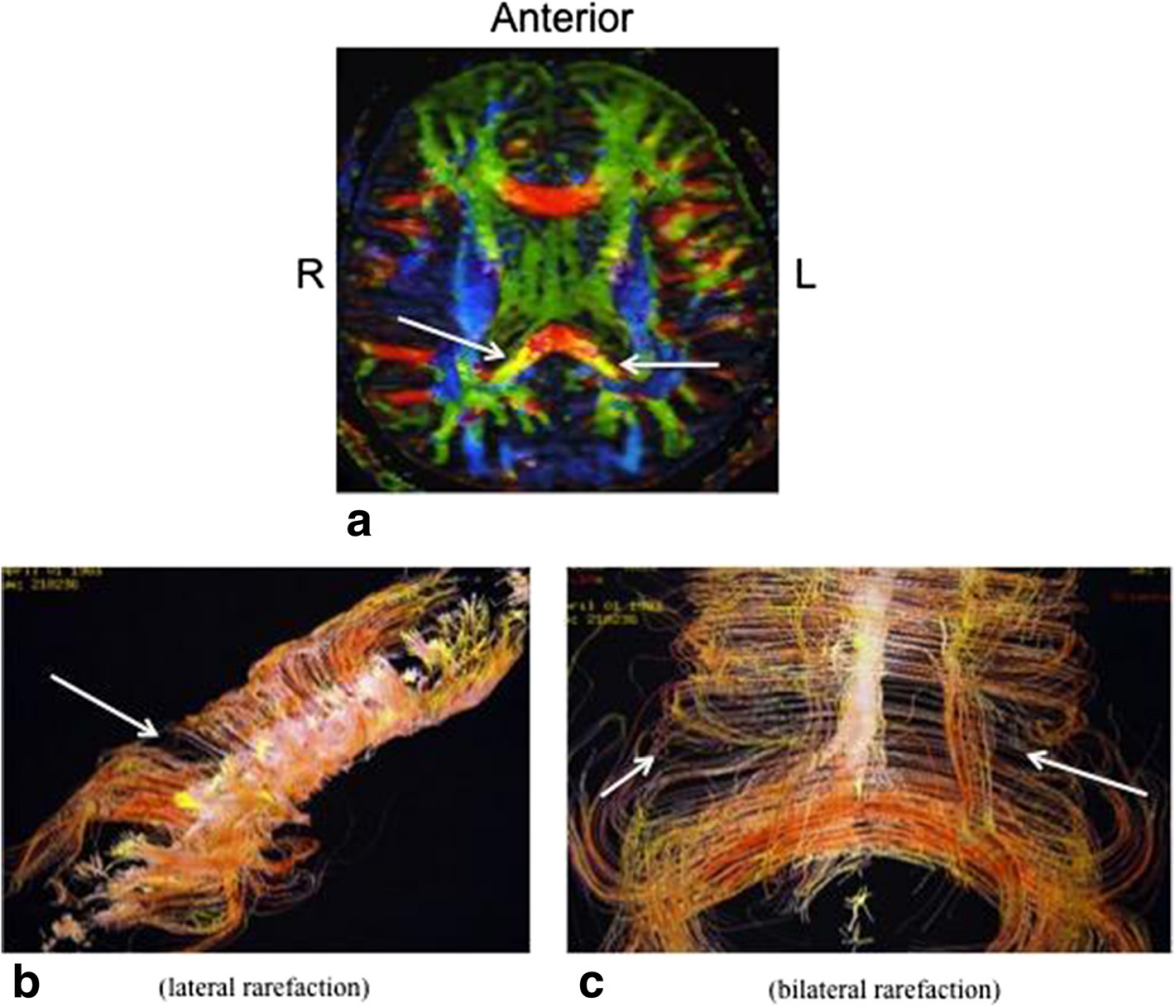
**a** Gloss drop of the corpus callosum. **b** and **c** Unilateral and bilateral rarefaction of white fibers in corpus callosum
- The fourth criterion was made of arcuate fasciculus (AF) injuries. Tridimensional-DTI typically showed white fibers fraying in this structure. White fibers located at the junction between the upper and posterior segments of the left AF were marked by a neat fraying out their normal path [Fig. 4ab]. In the case of that right-handed patient a shock also slightly sprayed out white fibers located at the posterior segment of her right arcuate fasciculus [Fig. 4cd]. Other possible patterns were that of a supple, elongated and finely structured aspect, especially in the right AF [Fig. 5abcd].

**Fig. 4:**
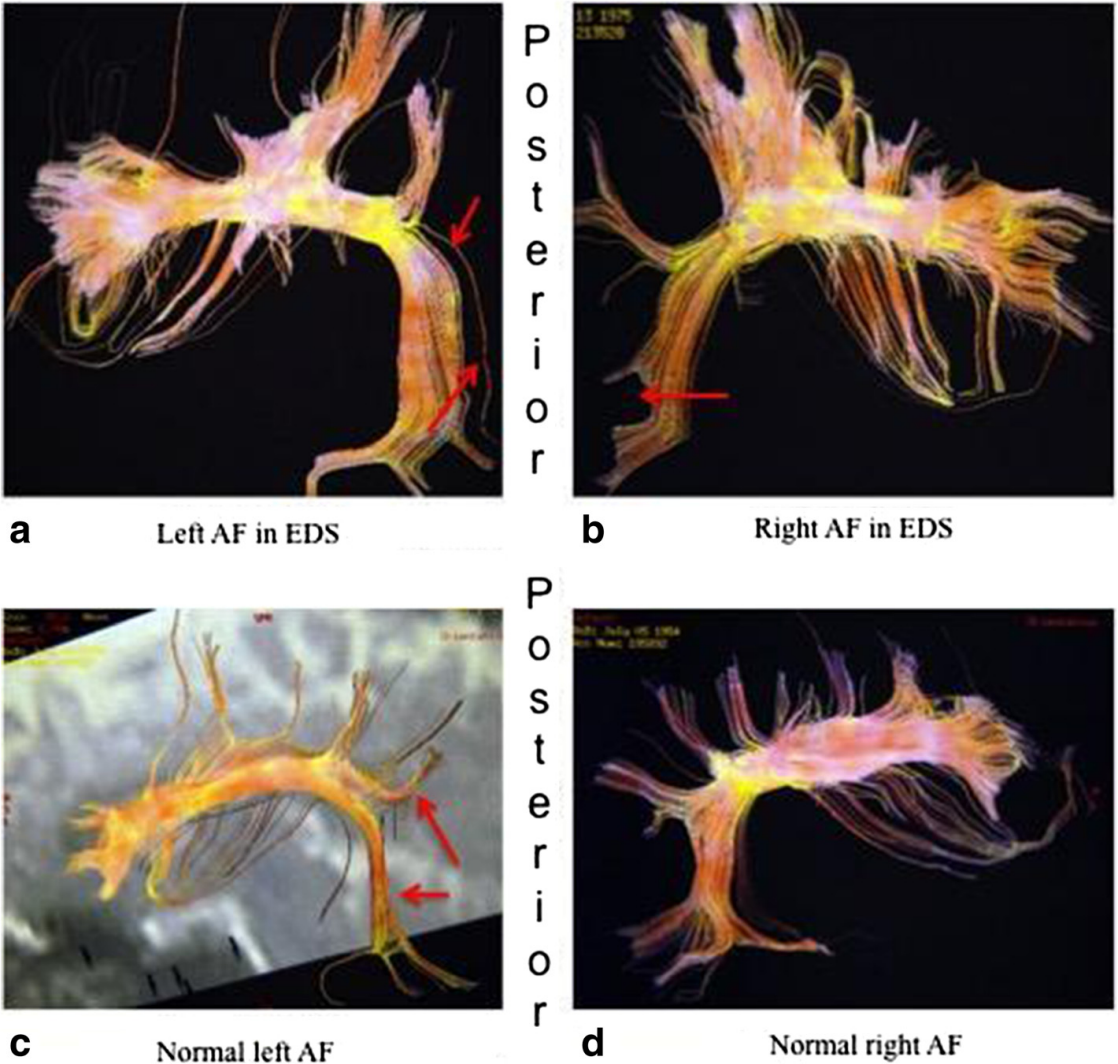
**a**,**b** White fibers fraying of the normal path of arcuate fasciculus. Those lesions were observed after a physical trauma (fall) and the affected women reported difficulties with her language, namely a low degree of dysphasia. **c**,**d** White fibers lesions located at the posterior segment of the right arcuate fasciculus

**Fig. 5:**
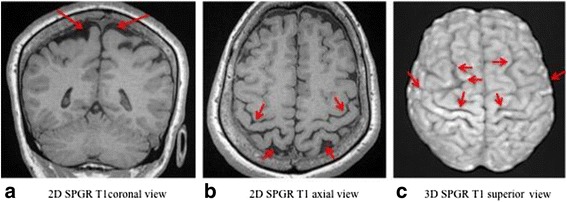
**a**,**b**,**c**,**d** Elongated and finely structured aspect of arcuate fasciculus
- The fifth retained criterion involved a bilateral sub-cortical atrophic aspect frequently observed at the following locations: retrocentral gyrus, anterior-superior internal portion of the parietal lobe, superior frontal sulcus displaying a marked bilateral abnormal width [Fig. 6abc].

**Fig. 6:**
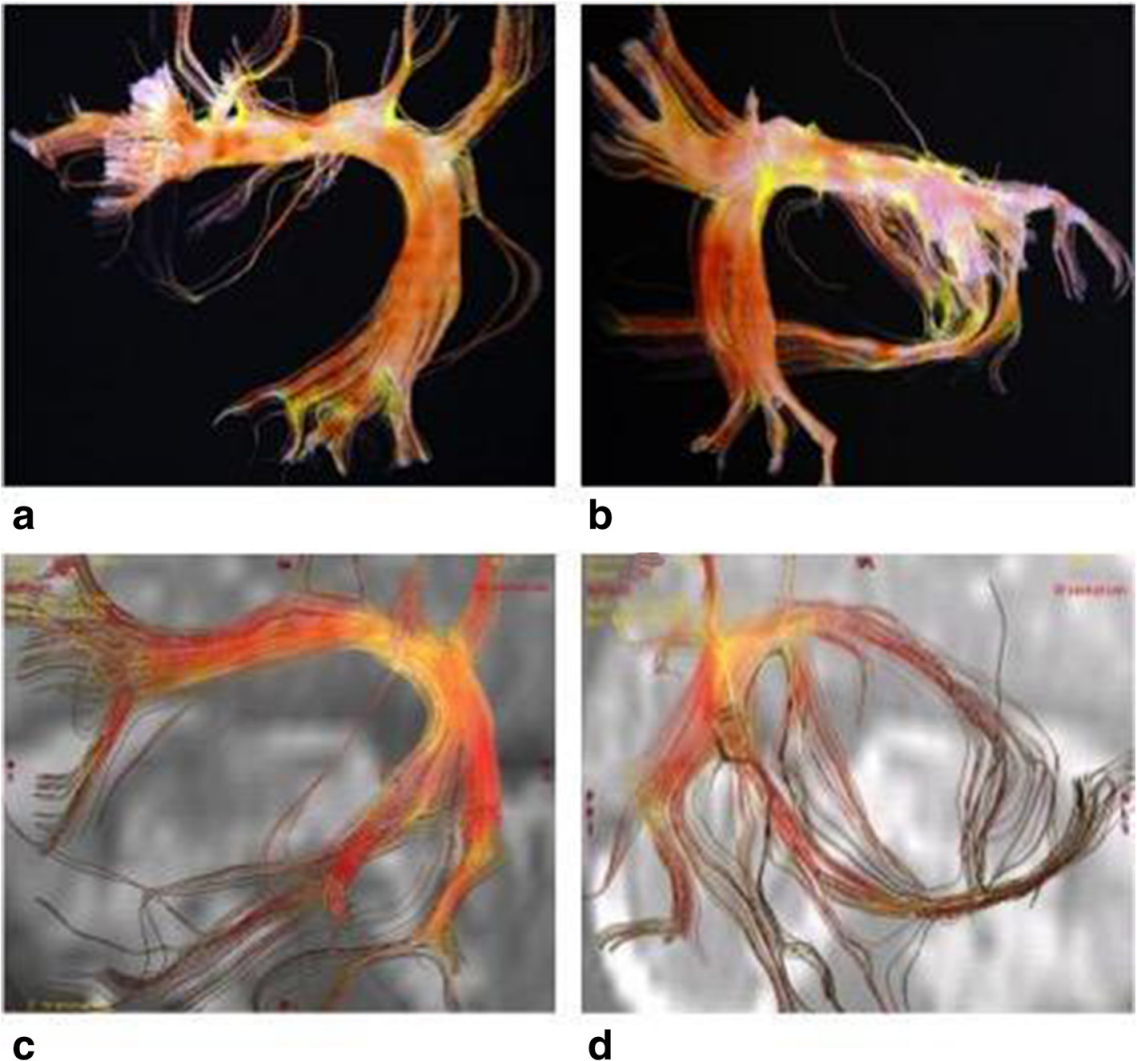
**a**,**b**,**c** Bilateral sub-cortical atrophic lesions of the retrocentral gyrus, anterior-superior internal portion of the parietal lobe and superior frontal sulcus
- The sixth criterion was an aspect of leucopathia, typical for its bilateral para-medial location presenting two strings of nodules made visible in axial hyperflash Flair sequence at the level of the two semi-oval hemi-centers [Fig. 7a and 7b

**Fig. 7:**
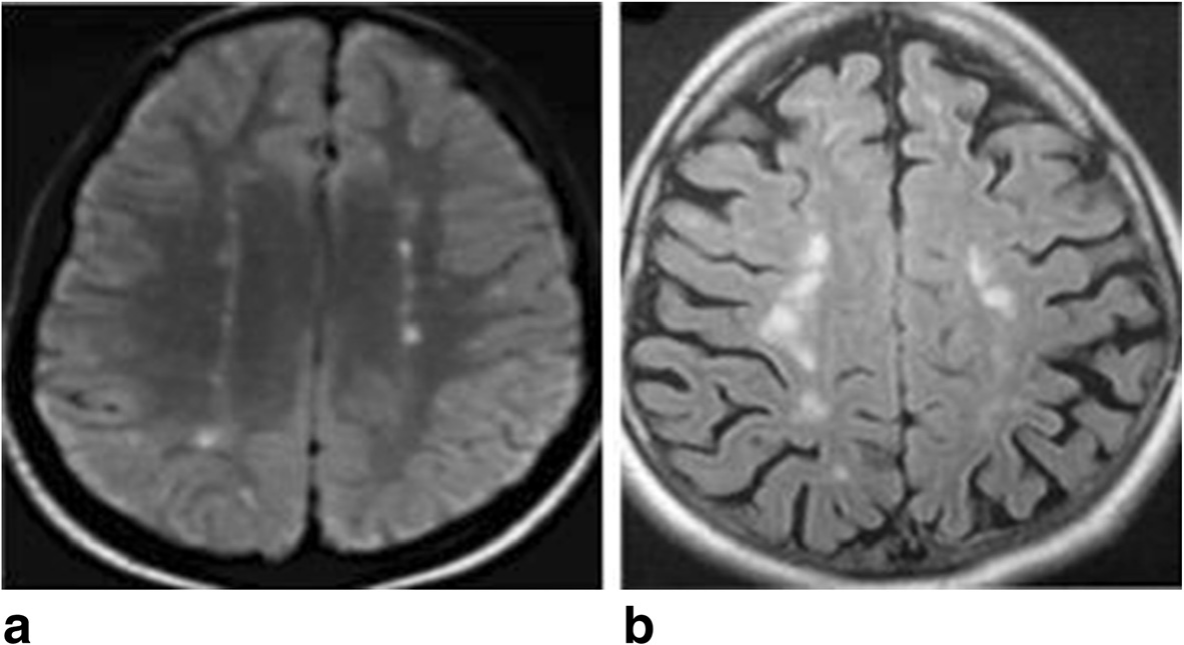
**a**,**b** Bilateral leucopathia

].

Actual prevalence of MRI findings in our cohort of patients is detailed in Table 1. The most frequent sign was the presence of lesion of the corpus callosum (98.3 %).

Overall, thirty-six patients (61.0 %) had at least 5 of the 6 imaging signs.

### Factors associated with the MRI findings

Women had more frequently injuries of the arcuate fasciculus (40/53; 75.5 % vs. 2/6; 33.3 %; *p* = 0.0308). Patients older than 40 had significantly more frequently leucopathia (8/28; 28.6 % vs. 2/31; 6.45 %; *p* = 0.0237). The laterality had no influence on the MRI findings.

The history of trauma was not associated with the presence of any of the six signs. However, lesions of the corpus callosum were observed in all of the 26 patients with a history of trauma. Moreover, in case of a trauma in childhood (before 18 years old), patients had significantly more lesions of the reticular formation (13/13; 100 % vs. 6/12; 50 %; *p* = 0.0035).

## Discussion

The present series of systematic cerebral imaging in patients with a hypermobility type of EDS showed the common prevalence of previously unreported brain lesions affecting white matter tracts. Importantly, near half of the patients of our series alleged a previous significant physical traumatism that could explain both the observed lesions and the worsening of several symptoms after their physical attack.

Our findings are novel, given that CNS lesions have only exceptionally been reported in EDS [10]. We categorized the observed lesions as six imaging signs that were thought to be sufficiently frequent and specific for claiming a high degree of validity. Some of those lesions had already been described by others and us but not in patients with EDS and foremost, not in such a consistent manner [11, 12]. The lesions that were herein identified and gathered systematically involved white matter tracts which are known to contain collagen fibers and this is thought to be consistent with established pathophysiology of EDS which is a paradigmatic collagen disorder [4]. Our findings provide an objective support to the definition and nosology of EDS, which could prove particularly helpful since clinical judgment remains the cornerstone for diagnosis and that genetic background is very incompletely known so far.

Foremost, we herein raise the hypothesis that the observed lesions could follow the physical traumas that were recorded in patients’ personal medical histories. The link between the trauma and the unmasking or worsening of EDS symptoms seems plausible for at least three reasons. First, although it is not widely recognized, some authors have described a certain degree of tissue fragility in EDS. It is therefore likely that any physical impact could provoke injuries, perhaps even potentially irreversible if one considers that the ability of connective tissue to heal is impaired in EDS. Second, many of our patients reported a clear temporal relationship between the trauma and exacerbation of their EDS symptoms. Last, most reported symptoms were thought to be consistent with the newly identified CNS lesions. Indeed, vigilance decrement, sleep disorders and fatigue can be related to the lesions observed of the reticular formation [13, 14]. Dystonia can plausibly be linked to the lesions of the two lenticular nuclei [15, 16].

Our study has limitations. First, our report is observational and causal link between observed lesions and clinical symptoms remains speculative. In effect, many of the alleged symptoms are subjective and non-specific. Moreover, those signs could be linked to serotoninergic medications even though our records do not suggest that those drugs were commonly taken by the patients of our cohort. Also, headaches are known to be common in EDS [17, 18] and we cannot state with certainty that the observed brain features were responsible for them. Last, one can always dispute the reliability of the diagnosis of EDS since there is currently no objective test to confirm the disease. However, our primary author has a great clinical expertise in the field and all patients were assessed through the commonly accepted clinical tools.

We believe our findings should prompt a debate in many respects regarding EDS. First, it should now be considered that some patients had their disease clinically induced or worsened by a physical trauma. This opens a way for compensation and many of our patients in the French national cohort are still struggling with Justice to obtain recognition of their prejudice. Second, further research, ideally with control groups, will be needed to confirm our findings and determine whether MRI could be considered as a possible tool in EDS assessment. Third, physical trauma should be prevented for those patients since we cannot exclude the risk of worsening of several subjective symptoms likely to impair their quality of life.

## Conclusions

We herein showed that patients with a hypermobility type of EDS were very frequently found to have specific brain lesions involving white matter tracts. Moreover, a significant proportion of our patients reported that a physical trauma had unmasked or worsened their EDS, suggesting that brain tissue characteristics explain the formation of those lesions following the alleged shock. One could imagine that the similar lesions observed in patients not remembering any trauma could be due to minor and forgotten injuries. In any case, our findings must be seen as a preliminary report that should propel more research on pathophysiology, imaging diagnosis and both preventive and therapeutic management regarding those patients.

### Ethics approval and consent to participate

This study was conducted in accordance with the French bioethics laws which do not require any approval by an ethic committee for clinical and imaging examinations as long as they are performed in the frame of standard cares. All patients provided written informed consent.

### Consent for publication

All patients gave informed consent for publication.

### Availability of data and material

Any material related to this work is available on request.

## Abbreviations

EDS: Ehlers-Danlos syndrome
MRI: magnetic resonance imaging
